# Vaccines Safety in Children and in General Population: A Pharmacovigilance Study on Adverse Events Following Anti-Infective Vaccination in Italy

**DOI:** 10.3389/fphar.2019.00948

**Published:** 2019-08-30

**Authors:** Niccolò Lombardi, Giada Crescioli, Alessandra Bettiol, Marco Tuccori, Marco Rossi, Roberto Bonaiuti, Claudia Ravaldi, Miriam Levi, Alessandro Mugelli, Silvia Ricci, Francesca Lippi, Chiara Azzari, Paolo Bonanni, Alfredo Vannacci

**Affiliations:** ^1^Department of Neurosciences, Psychology, Drug Research and Child Health, Section of Pharmacology and Toxicology, University of Florence, Florence, Italy; ^2^Tuscan Regional Centre of Pharmacovigilance, Florence, Italy; ^3^Unit of Adverse Drug Reactions Monitoring, Department of Clinical and Experimental Medicine, University of Pisa, Pisa, Italy; ^4^Centre of Pharmacovigilance, University Hospital of Siena, Department of Medical Sciences, Surgery and Neurosciences, University of Siena, Siena, Italy; ^5^CiaoLapo, Charity for Healthy Pregnancy, Stillbirth and Perinatal Grief Support, Prato, Italy; ^6^Department of Health Sciences, University of Florence, Florence, Italy; ^7^Epidemiology Unit, Department of Prevention, Local Health Unit Tuscany Centre, Florence, Italy; ^8^Department of Pediatric Immunology, University of Florence, Florence, Italy; ^9^Meyer Children’s Hospital, Florence, Italy

**Keywords:** vaccines, adverse events following immunization, pharmacovigilance, safety, observational study, preventability, seriousness

## Abstract

**Background:** The concern for adverse events following immunization (AEFI) and anti-vaccination movements that lacked scientific evidence-based supports may reduce vaccine uptake in the general population. Thus, the aims of the present study were to characterize AEFI in general population (all age groups), in terms of frequency, preventability, and seriousness and to define predictors of their seriousness in children.

**Methods:** A retrospective study was performed on suspected AEFI reports for children and adults who received any form of vaccinations, collected in Tuscany, Italy, between 1 January and 31 December 2017. Patients’ characteristics, suspected vaccines, and AEFI description were collected. Causality and preventability were assessed using WHO and Schumock and Thornton algorithms, respectively. Logistic regression was used to estimate the reporting odds ratios of potential predictors of AEFI seriousness in children.

**Results:** A total of 223 suspected AEFI reports were collected, and the majority of them were defined as non-serious (76.7%). Reports were mostly related to one vaccine, and to a median of two to five strains/toxoids. The total number of simultaneously administered strains/toxoids and the presence of allergens did not correlate with AEFI seriousness. Considering vaccines with a high number of administered doses (≥60,000 doses), the rates estimated for serious AEFI reports were always very low, ranging between 0.01 and 0.2/1,000 doses. Twenty-four vaccines (8,993 doses) were not related to any AEFI.

**Conclusion:** Results of present study showed that AEFI were very rare; the vast majority of them was non-serious and, despite the claims of anti-vaccination movements, the simultaneous administration of vaccines was safe and did not influence the risk of reporting a serious AEFI, particularly in children.

## Introduction

As vaccine-preventable infectious diseases continue to decline; parents and individuals in general have become increasingly concerned about the risks associated with vaccination. An adverse event following immunization (AEFI) is any untoward clinical occurrence which follows vaccinations and which does not necessarily have a causal relationship with vaccine use (Fulton et al., 2015; WHO, 2019a). An AEFI may be any unfavorable or unintended sign, abnormal laboratory finding, symptom, or disease. Moreover, AEFI classification may include: 1) vaccine product–related reaction (an AEFI that is caused or precipitated by a vaccine due to one or more of the inherent properties of the vaccine product), 2) vaccine quality defect–related reaction (an AEFI that is caused or precipitated by a vaccine that is due to one or more quality defects of the vaccine product including its administration device as provided by the manufacturer), 3) immunization-related errors, 4) immunization anxiety–related reactions, and 5) coincidental events (Fulton et al., 2015; WHO, 2019a). AEFI can be also represented by minor events, manifesting as local (i.e., erythema, edema, pain) or systemic (i.e., fever, exanthema, allergic reactions), and severe events manifesting as anaphylaxis, seizures, thrombocytopenia, hypotonic hypo-responsive episodes (HHE), or prolonged crying. Finally, an AEFI can be acute if it occurs within minutes after vaccine administration or delayed if it occurs several hours or days after vaccine administration (Danova et al., 2017). Considering the World Health Organization (WHO, 2019b) criteria, several considerations for assessing causality of an AEFI should be always evaluated: 1) temporal relationship, 2) alternative explanations, 3) proof of association, 4) prior evidence, 5) population-based evidence, and 6) biological plausibility. AEFI might be also related to strains/toxoids and/or to other vaccine components, such as: antibiotics (i.e., neomycin), components of the container (i.e., latex), residual proteins of the substrate (i.e., egg), or stabilizers (i.e., gelatine) (Autret-Leca et al., 2006).

In Italy, 18–25 AEFI reports are recorded every 100,000 vaccine doses. Overall, on a total of 4,766 AEFI reports collected during 2016 at the National level, more than 80% were defined as non-serious (AIFA, 2018). Nevertheless, in the last decade, public health authorities worldwide have observed an increase in the number of vaccine hesitant patients or parents (Larson et al., 2016) and an increase in “anti-vaccination” movements activities (Tafuri et al., 2015). The concern for vaccine safety and dearth of studies documenting surveillance of their adverse effects were the major theme of anti-vaccination movements (Tafuri et al., 2015). As a consequence, immunization coverage in Italy has decreased both in children (Signorelli et al., 2017) and adults (Pezzotti et al., 2018) in recent years. Compulsory vaccination for children, therefore, became enacted into Law in July 2017 (Law n. 119) in order to address the vaccination short fall in Italy. Consequently, the number and uptake of compulsory vaccination have increased from 4 to 10 (Scavone et al., 2018b). These are those included in the hexavalent vaccine (*diphtheria, tetanus, pertussis, poliomyelitis*, hepatitis B, and *hemophilus influenzae B*) and measles, mumps, rubella, and varicella, for children up to 6 years of age to be enrolled in kindergartens and pre-schools and for children up to 16 years of age to attend compulsory schools. Furthermore, health authorities are requested to promote all vaccines (mandatory and non-mandatory) recommended in the 2017–2019 National Immunisation Plan (Scavone et al., 2018b). As a matter of fact, vaccines such as those against *Neisseria meningitidis*, *Streptococcus pneumoniae*, or human papilloma virus are not included in the mandatory vaccination law; however, they are included in the recent Italian National Vaccination Plan (2015–2017) and are given free of charge to all newborns (*Neisseria meningitidis* and *Streptococcus pneumoniae*) or to all adolescents, both male and female (HPV vaccine).

Thus, improving AEFI characterization in the general and pediatric population remains a public health and a patient safety perception priority, especially after the new regulatory decision. Moreover, to the best of our knowledge, no studies have been previously conducted to assess the possible association between vaccines’ characteristics (number of strains/toxoids simultaneously administered and presence of allergens as reported in the summary of product characteristic, SPC) and AEFI reporting.

The aim of the present study was to characterize AEFI in the general population evaluating their frequency in relation to the total number of administered doses, their preventability, and seriousness. Moreover, we also identified predictors of AEFI seriousness in children, by means of 1-year pharmacovigilance study.

## Material and Methods

This is a retrospective study performed on reports of suspected AEFI collected in Tuscany, Italy, between 1 January and 31 December, 2017, and related only to vaccinations performed during such period. We analyzed all suspected AEFI reported from patients having at least one AEFI. All AEFI reports were collected through both the regional spontaneous reporting system and two active pharmacovigilance projects, *Monitoring of the Adverse Effects in Pediatric population* (MEAP) (Carnovale et al., 2014) and *Epidemiological Monitoring of Adverse Drug Reactions in Emergency Department* (MEREAFaPS) (Lombardi et al., 2018).

Each AEFI report demographic characteristics, including age, sex, and ethnic group, were recorded. Anatomical Therapeutic Chemical (ATC) classification system was used to code both vaccine administered and concomitant medications, for which we also recorded the administration route, therapy duration, and dosage. The description of AEFI according to diagnosis and symptoms was coded using the Medical Dictionary for Regulatory Activities (MedDRA) and organized by System Organ Class (SOC) (Lombardi et al., 2018). Seriousness of AEFI was classified according to the World Health Organization (WHO) criteria (Lombardi et al., 2018) as fatal, life-threatening, requiring hospitalization of the patient, or causing serious/permanent disability.

The most frequently reported SOC and ATC classes were also evaluated. For each AEFI report, causality (categorized as *consistent*, *inconsistent*, *indeterminate*, or *unclassifiable*) and preventability (categorized as *definitely* or *probably* *preventable*, or *not* *preventable*) were assessed using the WHO algorithm (Tozzi et al., 2013; WHO, 2018) and Schumock and Thornton algorithm (Schumock and Thornton, 1992), respectively. Both assessments were performed by a multidisciplinary team composed by experts in clinical pharmacology, immunology, epidemiology, and pediatrics. The same team revised data on concomitant medications and analyzed vaccine–vaccine (VVI), vaccine-drug (VDI), and drug–drug (DDI) interactions using the drug interaction software Micromedex Drug-REAX System (Thomson Reuters Healthcare Inc., Greenwood Village, Colorado, United States), available online with restricted access (Santos et al., 2017). As reported in the Micromedex tool (Micromedex® 2.0, 2013), interactions were classified as mild, moderate, or severe, depending on their clinical complexity.

For each vaccine, the number of bacterial or viral strains and toxoids was obtained from the specifications listed in the SPC. In case of multiple vaccines’ administration in the same vaccination session, we obtained the total amount of strains/toxoids administered by summing the number of strains/toxoids for each product. Allergens listed in the SPC were considered together as a dichotomous variable (presence/absence). We considered the presence/absence of: neomycin and/or other antibiotics in traces, latex, egg, gelatine, etc.

For the general population (all age groups), the frequency of total and serious AEFI was calculated in relation to the total number of vaccines doses administered in Tuscany, Italy, in 2017. AEFI rates were calculated dividing the number of AEFI reports by the total number of administered doses for each ATC of interest and expressed as *AEFI reports/administered doses x 1,000*. Data regarding AEFI reports and vaccines administered doses were obtained by the Regional Health Authority, after having been made anonymous. Due to the lack of information on total administered doses for influenza vaccines, all ATC classes related to these products were not included in the analysis, and AEFI rates for them were not calculated. AEFI reports potentially associated with those vaccines were nevertheless collected, assessed, and discussed.

For subjects ≤18 years, predictors of AEFI seriousness, such as age groups, sex, number of vaccines simultaneously administered, vaccines characteristics (number of strains/toxoids and presence of allergens), and interactions (DDIs, VDIs, VVIs), were also evaluated. For children, age groups were codified using Food and Drug Administration (FDA) classification (FDA, 2014).

Descriptive statistics were used to summarize data. Categorical data were reported as frequencies and percentages and compared using the chi-square or Fischer exact tests, as appropriate, whereas continuous data were reported as median values with the related interquartile ranges (IQR) and compared using the Mann-Whitney test. Univariate and multivariate logistic regressions were used to estimate the reporting odds ratios (RORs) with 95% confidence intervals (CIs) of potential predictors of AEFI seriousness in children. ROR values for each variable were reported both as crude and adjusted values and adjusted for all the other considered variables. All results were considered to be statistically significant at *p < 0.05*. Data management and statistical analysis were carried out using STATA 14.

The coordinating center of Tuscany, Italy, approved MEREAFaPS project (*notification number 1225—December 21, 2009*), and the local institutional ethics committee approved MEREAFaPS (*study number 3055/2010, protocol number 45288—August 6, 2014*) according to the legal requirements concerning observational studies. The MEAP project was approved in the frame of MEREAFaPS. Due to the retrospective nature of the present study and data anonymization, patient’s consent to participate was not required.

## Results

In 2017, the entire population of Tuscany included around 3,736,968 inhabitants, of which 601,160 (16%) were children. Within the study period, a total of 223 AEFI reports were collected and evaluated; of them, 171 (76.7%) were defined as non-serious, and 52 (23.3%) were defined as serious. **Table 1** shows the characteristics of all suspected AEFI reports (on the top of **Table 1**) and the characteristics of each AEFI (on the bottom of **Table 1**) in the general population.

**Table 1:** Characteristics of all AEFI reports (top) and each AEFI (bottom) in general population.

	Tot reports n = 223 (%)	Non-serious n = 171 (%)	Serious n = 52 (%)	p-Value
**N AEFI for each report**				
1	76 (34.08)	57 (33.33)	19 (36.54)	*0.503*
2	43 (19.28)	35 (20.47)	8 (15.38)	
3	49 (21.97)	40 (23.39)	9 (17.31)	
4+	55 (24.66)	39 (22.81)	16 (30.77)	
**Age, years (overall)**				
Median (IQR)	9.24 (1.14–42.17)	9.95 (1.24–44.25)	2.26 (0.84–22.28)	*0.091*
**Pediatric age groups (FDA classification)**				
Newborns (< 1 month)	0	0	0	*0.664*
Infants (1 month–2 years)	57 (25.56)	40 (23.39)	17 (32.69)	
Children (2–12 years)	73 (32.74)	56 (32.75)	17 (32.69)	
Adolescents (12–18 years)	12 (5.38)	8 (4.68)	4 (7.69)	
**Adults (quartiles of age)**				
19–39 years	20 (8.97)	16 (9.36)	4 (7.69)	
40–49 years	21 (9.42)	18 (10.53)	3 (5.77)	
50–59 years	15 (6.73)	13 (7.60)	2 (3.85)	
Over 60 years	25 (11.21)	20 (11.70)	5 (9.62)	
**Sex**				
Female	102 (45.74)	84 (49.12)	18 (34.62)	*0.036*
Male	114 (51.12)	80 (46.78)	34 (65.38)	
*Missing*	7 (3.14)	7 (4.09)	0	
**Ethnic group**				
Caucasian	137 (61.43)	106 (61.99)	31 (59.62)	*0.880*
Others	14 (6.28)	10 (5.85)	4 (7.69)	
*Missing*	72 (32.29)	55 (32.16)	17 (32.69)	
**Causality**				
Consistent	153 (68.61)	129 (75.44)	24 (46.15)	* <0.001*
Inconsistent	24 (10.76)	7 (4.09)	17 (32.69)	
Indeterminate	44 (19.73)	33 (19.30)	11 (21.15)	
Unclassifiable	2 (0.90)	2 (1.17)	0	
Preventability				
No	207 (92.80)	162 (94.74)	45 (86.53)	*0.016*
Yes	16 (7.20)	9 (5.26)	7 (13.47)	
	**Tot AEFI** **n = 570**	**Non-serious** **n = 431**	**Serious** **n = 139**	**p-Value**
**Number of suspect drug for each AEFI**				
1	489 (85.79)	379 (87.94)	110 (79.14)	*0.002*
2	74 (12.98)	45 (10.44)	29 (20.86)	
3	7 (1.23)	7 (1.62)	0	
**Concomitant drugs (not suspected)**				
0	502 (88.07)	383 (88.86)	119 (85.61)	*0.023*
1	35 (6.14)	22 (5.10)	13 (9.35)	
2	23 (4.04)	21 (4.87)	2 (1.44)	
3	9 (1.58)	5 (1.16)	4 (2.88)	
4	1 (0.18)	0	1 (0.72)	
**Tot strains/toxoids**				
Median (IQR)	4 (4-7)	4 (3-7)	4 (4-6)	*0.815*
1	51 (8.95)	36 (8.35)	15 (10.79)	*0.940*
2–5	364 (63.86)	277 (64.27)	87 (62.59)	
6–9	53 (9.30)	40 (9.28)	13 (9.35)	
10–13	26 (4.56)	20 (4.64)	6 (4.32)	
14+	76 (13.33)	58 (13.46)	18 (12.95)	
**Presence of allergens (in traces)**				
Yes	222 (38.95)	180 (41.76)	42 (30.22)	*0.015*
No	348 (61.05)	251 (58.24)	97 (69.78)	
**Seriousness (out of 139)**				
Death	0	-	0	-
Persistent or significant disability/incapacity	8 (5.76)	-	8 (5.76)	
Congenital abnormalities/birth defects	0	-	0	
Hospitalization or prolongation	83 (59.71)	-	83 (59.71)	
Life-threatening condition	0	-	0	
Other clinically relevant conditions	48 (34.53)	-	48 (34.53)	
**Outcome**				
Complete resolution	247 (43.33)	184 (42.69)	63 (45.32)	* <0.001*
Improvement	119 (20.88)	98 (22.74)	21 (15.11)	
Still unresolved^#^	78 (13.68)	50 (11.60)	28 (20.14)	
Resolution with sequelae	5 (0.88)	5 (1.16)	0	
Unchanged/worsened reaction	2 (0.35)	2 (0.46)	0	
Death	5 (0.88)	0	5 (3.60)	
*Missing°*	114 (20.00)	92 (21.35)	22 (15.83)	
**Interactions (DDIs + VDIs + VVIs)**				
Not applicable (one treatment)	450 (78.95)	346 (80.28)	104 (74.82)	*0.032*
No	106 (18.60)	78 (18.10)	28 (20.14)	
Mild	0	0	0	
Moderate	2 (0.35)	2 (0.46)	0	
Severe	12 (2.11)	5 (1.16)	7 (5.04)	

IQR, interquartile range; FDA, Food and Drug Administration; DDI, drug–drug interaction; VDI, vaccine-drug interaction; VVI, vaccine–vaccine interaction.

^#^From a case-by-case evaluation these events were classified as still unresolved due to the fact that the AEFI reporting was performed immediately after AEFI onset.

°These events were classified as missing because the reporter did not complete the relative section present in the AEFI reporting form.

p-value was from Fisher Exact test for percentages and Mann-Whitney test for median values.

### Characteristics of AEFI Reports

The median age was 9.24 years (IQR, 1.14–42.17), and children (n = 142, 63.7%) experienced more AEFI than adults. However, there was no statistically significant difference among age groups when serious and non-serious AEFI reports were compared (p = 0.664). We encountered more AEFI reports referred to males than females, and the distribution of the type of events according to sex was significantly different (p = 0.036). AEFI reports were more common among Caucasians.

Evaluating the causality assessment, 153 AEFI reports (68.6%) were defined as consistent with the vaccination, 24 (10.8%) as inconsistent, 44 (19.7%) as indeterminate, and 2 (0.9%) were defined as unclassifiable. Applying the Schumock and Thornton algorithm, more than 90% of suspected AEFI reports were classified as non-preventable, while 16 reports were defined as preventable.

In particular, within preventable AEFI reports, we encountered: six cases of major VDIs observed between *Pneumococcus, purified polysaccharides antigen-conjugated* (ATC J07AL02), and acetaminophen; one case of moderate VDI observed between *varicella, live-attenuated* (ATC J07BK01), and betamethasone; one case of moderate VVI observed between *Meningococcus C, purified polysaccharides antigen-conjugated* (ATC J07AH07), and *diphtheria-hemophilus influenzae B-pertussis-poliomyelitis-tetanus-hepatitis B* (ATC J07CA09); three cases of post-immunization hypersensitivity reactions in patients with a previous history of allergy; three cases of patients who developed the same AEFI experienced following the administration of previous doses for the same vaccine (positive rechallenge); one case of therapeutic error; and one case of AEFI report observed following *live-attenuated rotavirus* (ATC J07BH01) administration in a patient with a previous history of intestinal intussusception.

### Characteristics of AEFI

Regarding each AEFI (n = 570), the majority of them was associated with only 1 vaccine (n = 489, 85.8%), and 139 (24.4%) were defined as “serious.” Of the 139 serious AEFI reported in this study, 83 (59.7%) resulted in hospitalization, and 48 were represented by “other clinically relevant conditions” (i.e., all adverse events judged by the reporter as clinically relevant because they required an intervention to prevent one of the other characteristics and consequences related to death, or life threatening, or hospitalization, or permanent disability, etc.). “Hospitalization or prolongation” group was mostly associated with vaccine-related fever, and within “other clinically relevant conditions” group; only two cases of death (for a total of five vaccine-AEFI pairs) were observed.

These fatal cases involved two women, aged 68 and 97 years old (data not shown in **Table 1**), who died for an acute respiratory tract infection (suspected vaccine ATC J07AL02) and for a cardiac failure in hypertensive cardiopathy (suspected vaccine ATC J07BB02), respectively. After an accurate clinical evaluation, these fatal cases were judged as not associated to vaccination. Finally, we encountered eight vaccine-AEFI pairs (derived from two AEFI reports) associated to “persistent or significant disability/incapacity” group, which were reported by patient/citizen and, consequently, misclassified. In fact, the clinical evaluation performed by the panel of experts revealed that these AEFI had indeed a complete resolution and were not causally consistent with vaccination.

Regarding the outcome, 366 (64.2%) AEFI were completely resolved or improved, and 78 (13.7%) were still unresolved at the time of reporting. The outcome was missing for 114 (20%) AEFI. Finally, the outcome of five (0.9%) AEFI was defined as resolution with sequelae and referred to a single patient for whom the observed events were not associated to the vaccination. Of note, as mentioned above, for a total of five vaccine-AEFI pairs (0.9%) classified within the “other clinically relevant conditions” group and associated with the outcome death, the clinical evaluation revealed that these events were not causally correlated with vaccination.

Out of a total of 120 AEFI, only 14 of them presented a moderate (n = 2) or severe (n = 12) interaction. These interaction-related AEFI derived from eight AEFI reports (seven cases of VDIs, one case of VVI) described above in the section of reports’ characteristics.

Following MedDRA hierarchy, 267 (46.8%) out of 570 AEFI belonged to “general disorders and administration site conditions,” 67 (11.7%) involved “nervous system disorders,” 62 (10.9%) involved “skin and subcutaneous tissue disorders,” 60 (10.5%) involved “gastrointestinal disorders,” and 30 (5.3%) involved “musculoskeletal disorders.” Consequently, the five most frequently reported preferred terms were: fever (17.5%), pain at site of vaccination (5.3%), rash at site of vaccination (5.3%), rash at unspecified site (5.1%), and edema at site of vaccination (4.7%) (**Supplementary Table 1**). Of note, within the SOC “nervous system disorders,” only six cases of febrile-related seizures were reported, all of them with a complete resolution.

**Supplementary Table 2** shows the characteristics of all suspected AEFI reports (on the top) and the characteristics of each AEFI (on the bottom) observed for pediatric population, described taking into consideration the current National Vaccination Plan 2017–2019. As already reported for the general population, we observed similar results also for the pediatric one.

### Administered Doses and AEFI Reporting

Considering all vaccines doses administered in Tuscany, we observed very few AEFI reports for vaccines with a high frequency of usage (more than 60,000 doses). In particular, as reported in **Table 2**, the most frequently administered vaccines were: *Meningococcus B, multicomponent vaccine* (117,525 doses); *Meningococcus C, purified polysaccharides antigen-conjugated* (109,515 doses); *Pneumococcus, purified polysaccharides antigen-conjugated* (72,273 doses); *diphtheria-hemophilus influenzae B-pertussis-poliomyelitis-tetanus-hepatitis B* (65,158 doses); and *Meningococcus A, C, Y, and W-135, tetravalent purified polysaccharides antigen-conjugated* (61,590 doses). For them, the rates of total and serious AEFI reports per 1,000 administered doses were very low and varied from 0.07 to 0.54, and from 0.01 to 0.20, respectively. Several vaccines were not associated with any AEFI. **Supplementary Table 3** reports the number of administered doses of all ATC classes for which no AEFI reports were collected during the study period.

**Table 2:** Number of administered doses and rates of AEFI reports in general population.

Vaccine	ATC	N strains/toxoids	N AEFI report total	N AEFI report serious	N administered doses	Rate of total AEFI reports per 1,000 doses	Rate of serious AEFI reports per 1,000 doses
Meningococcus B, multicomponent vaccine	J07AH09	4	63	23	117525	0.54	0.20
Meningococcus C, purified polysaccharides antigen-conjugated	J07AH07	2	8	1	109515	0.07	0.01
Pneumococcus, purified polysaccharides antigen-conjugated	J07AL02	14	30	8	72273	0.42	0.11
Diphtheria-hemophilus influenzae B-pertussis-poliomyelitis-tetanus-hepatitis B	J07CA09	10	18	2	65158	0.28	0.03
Meningococcus A, C, Y, and W-135 tetravalent purified polysaccharides antigen-conjugated	J07AH08	5	27	2	61590	0.44	0.03
Pertussis, purified antigen, combinations with toxoids	J07AJ52	5	9	2	55478	0.16	0.04
Measles, combinations with mumps, rubella and varicella, live-attenuated	J07BD54	4	14	4	45989	0.30	0.09
Diphtheria-pertussis-poliomyelitis-tetanus	J07CA02	8	11	1	44188	0.25	0.02
Papillomavirus (human types 16, 18)	J07BM02	4	4	0	21179	0.19	0.00
Measles, combinations with mumps and rubella, live-attenuated	J07BD52	3	23	8	18837	1.22	0.42
Tetanus toxoid, combinations with diphtheria toxoid	J07AM51	2	4	0	13598	0.29	0.00
Varicella, live-attenuated	J07BK01	1	4	1	13351	0.30	0.07
Tetanus toxoid	J07AM01	1	9	2	12669	0.71	0.16
Hepatitis B, purified antigen	J07BC01	1	6	1	8826	0.68	0.11
Hepatitis A, inactivated, whole virus	J07BC02	1	1	0	8803	0.11	0.00
Rota virus, live-attenuated	J07BH01	1	6	2	8499	0.71	0.24
Rota virus, pentavalent, live, reassorted	J07BH02	5					
Typhoid, oral, live-attenuated	J07AP01	1	2	1	3045	0.66	0.33
Poliomyelitis oral, trivalent, live-attenuated	J07BF02	3	1	0	2137	0.47	0.00
Poliomyelitis, trivalent, inactivated, whole virus	J07BF03	3					
Poliomyelitis oral, bivalent, live-attenuated	J07BF04	2					
Cholera, inactivated, whole cell	J07AE01	5	1	1	1495	0.67	0.67
Papillomavirus (human types 6, 11, 16, 18, 31, 33, 45, 52, 58)	J07BM03	9	1	1	793	1.26	1.26
Rabies, inactivated, whole virus	J07BG01	1	1	0	439	2.28	0,00
Pneumococcus, purified polysaccharides antigen	J07AL01	23	2	1	351	5.70	2.85
Diphtheria-hemophilus influenzae B-pertussis-poliomyelitis-tetanus	J07CA06	8	1	1	152	6.58	6.58

### Predictors of AEFI Seriousness in Children

**Table 3** shows demographic and clinical features associated with the risk of serious AEFI reporting in the pediatric age classes specifically mentioned in the National Vaccination Plan 2017–2019. Male sex and the presence of concomitant (not suspected) drugs were associated with a significantly higher risk of AEFI reported as serious. The total amount of strains/toxoids administered and the presence of allergens were not associated with AEFI seriousness, for all evaluated age groups.

**Table 3:** Association between serious AEFI risk and different factors expressed as reporting odds ratio (ROR) within pediatric population stratified according to the age classes of the National Vaccination Plan 2017–2019.

	0–15 months		16 months–12 years		12–18 years	
	Adjusted ROR (95% CI)	p-Value	Adjusted ROR (95% CI)	p-Value	Adjusted ROR (95% CI)	p-Value
**Sex**						
Female	Ref.					
Male	1.26 (0.48–3.27)	0.639	2.29 (1.10–4.76)	0.027	0.20 (0.02–2.39)	0.203
**Concomitant drugs (not suspected)**						
No	Ref.					
Yes	3.20 (0.96–10.70)	0.059	6.88 (1.42–33.43)	0.017	–	–
**Tot strains/toxoids**						
1–6	Ref.					
6+	0.16 (0.02–1.30)	0.087	2.14 (0.97–4.74)	0.060	–	–
**Presence of allergens (in traces)**						
No	Ref.					
Yes	0.31 (0.04–2.56)	0.280	0.94 (0.45–1.98)	0.873	–	–

### Distribution of AEFI Reports

**Figure 1:**
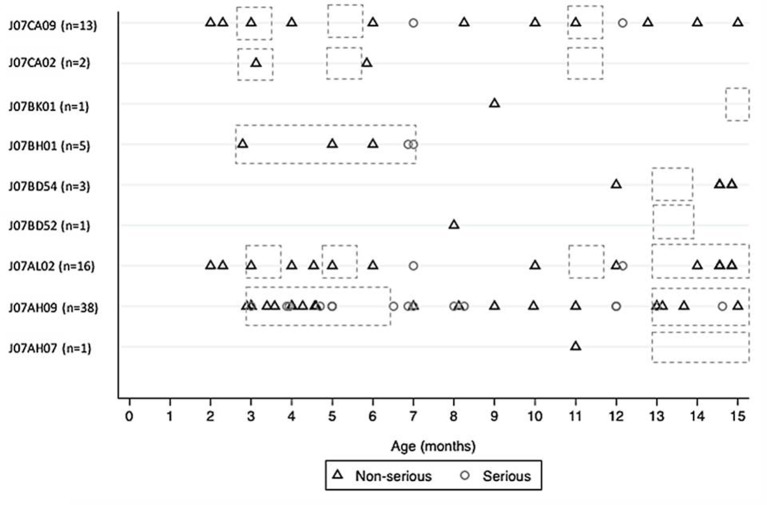
Distribution of AEFI reports (non-serious and serious) collected for mandatory and non-mandatory vaccinations performed between 0 and 15 months of age, according to the National Vaccination Plan 2017–2019 (dashed areas). **Mandatory: “**Diphtheria-hemophilus influenzae B-pertussis-poliomyelitis-tetanus-hepatitis B” (J07CA09); “Diphtheria-pertussis-poliomyelitis-tetanus” (J07CA02); “varicella, live-attenuated” (J07BK01); “measles, combinations with mumps, rubella and varicella, live-attenuated” (J07BD54); “measles, combinations with mumps and rubella, live-attenuated” (J07BD52). **Non-mandatory:** “Rotavirus, live-attenuated” (J07BH01); “Pneumococcus, purified polysaccharides antigen-conjugated” (J07AL02); “Meningococcus B, multicomponent vaccine” (J07AH09); “Meningococcus C, purified polysaccharides antigen-conjugated” (J07AH07).

**Figure 1** describes the distribution of AEFI reports collected for mandatory (J07CA09, J07CA02, J07BK01, J07BD54, J07BD52) and non-mandatory (J07BH01, J07AL02, J07AH09, J07AH07) vaccinations performed between 0 and 15 months of age, according to the National Vaccination Plan 2017–2019. For other vaccinations (mandatory and non-mandatory), no AEFI reports were collected in children aged 0–15 months. Dashed areas represents the age period when vaccination should be administered. The majority of AEFI reports (non-serious and serious) occurred within the time window for vaccine administration, demonstrating that vaccinations, were administered strictly following health authority dispositions. Some non-serious AEFI occurred before or after the recommended time window for vaccination, in particular to those related to *Meningococcus B, multicomponent vaccine* (J07AH09), and *diphtheria-hemophilus influenzae B-pertussis-poliomyelitis-tetanus-hepatitis B* (J07CA09). Data related to the other age groups are shown in the **Supplementary Table 4**.

## Discussion

This study aimed to characterize AEFI in the general population, evaluating their frequency in relation to the total number of administered doses and their preventability and seriousness, and to identify predictors of AEFI seriousness in children. To the best of our knowledge, this is the first study considering vaccine characteristics, in particular to the number of strains/toxoids simultaneously administered and the presence of allergens, as factors potentially associated to AEFI seriousness in patients aged ≤18 years. Moreover, our study was performed in the light of the last Italian National Vaccination Plan 2017–2019.

In terms of immunization safety, our study showed a higher frequency of non-serious AEFI reports, both in the general and in the pediatric population. Moreover, the majority of the events were not preventable, highlighting the appropriateness of vaccines use and administration in the observed population. In fact, the specific algorithm used to assess AEFI preventability contains a section which helps the assessor to evaluate whether or not an AEFI is related to an inappropriate use of the vaccine (Schumock and Thornton, 1992). In particular, the answer “NO” to the points 2 and 3 (Section A) of the algorithm indicates a high level of appropriateness of vaccination. Most of the preventable AEFI reports were due to the potential interaction between the components of the vaccines or between the vaccines and concomitant medications administered to the patients. Previous studies have documented poor immunologic response to conjugated *Pneumococcus* vaccine following acetaminophen administration (Prymula et al., 2009; Falup-Pecurariu et al., 2017; Wysocki et al., 2017). We also reported one case of interaction between *varicella, live-attenuated* vaccine, and the glucocorticoid betamethasone, which may result in an inadequate immunological response to the live vaccine (Infectious Diseases and Immunization Committee, Canadian Paediatric Society, 2000; Liu et al., 2013). Finally, we collected a VVI reported between *Meningococcus C, purified polysaccharides antigen-conjugated* vaccine, and *diphtheria-hemophilus influenzae B-pertussis-poliomyelitis-tetanus-hepatitis B* vaccine. The administration of *Meningococcal groups A, C*, and *W-135 and Y conjugate vaccine* after the *Tetanus toxoid* vaccine may result in lower geometric mean titers against groups A, C, and W-135 (Rivera et al., 2018). In particular, the present interaction was observed in a 2-year old female who received the two vaccinations out of the time window indicated for the administration of these vaccines, both in the SPC and in the National Vaccination Plan 2017–2019.

In our study, the most common AEFI related to vaccines were fever, injection-site reactions, rash, vomit, and fatigue, i.e., mild and transient untoward medical occurrences. Our results are comparable to those already published in literature, both in adults (Alguacil-Ramos et al., 2016; Mentzer et al., 2018) and in children (Danova et al., 2017; Lopes et al., 2018; Mentzer et al., 2018). We also reported six cases of febrile-related seizures (i.e., 1.05% of AEFI). Although all of them were classified as serious, since they caused an emergency department (ED) admission, a complete resolution was reported in all cases. The majority of vaccines can cause fever, and some children who develop a fever can have a seizure. However, studies showed there is one case of fever-triggered seizure for every 3,000 to 4,000 children who receive a vaccine (Marin et al., 2010), and these seizures almost never cause harm over the long term (Committee to Review Adverse Effects of Vaccines, 2011). Considering AEFI-related ED visits or hospitalizations, the majority of them were referred to the preferred terms “fever” and “hyperpyrexia,” adverse events well known and well characterized after vaccination (Sawyer et al., 2016).

Considering the high number of vaccine doses administered in Tuscany in 2017 (more than 600,000 doses), the total number of vaccine-related AEFI detected in the present study is low and provides a useful documentation of vaccination safety, both in adults and in children (Staltari et al., 2013). Notably, in terms of causality, no death was associated with vaccination. In fact, regarding the first case of decease, the suspected vaccine was ATC J07AL02 (*Pneumococcus, purified polysaccharides antigen-conjugated*), but clinical examination revealed that lung infection was attributable to a pneumococcal strain different from those contained in the 13-valent vaccine, excluding a vaccination failure. In the second case of death, suspected vaccine was ATC J07BB02 (*influenza, inactivated, split virus, or surface antigen*), which caused a fever-related ED visit, but the decease occurred 15 days later and was related to a pre-existent heart failure.

Concurrent administration of vaccines with multiple strain/toxoid was not a predictor of serious AEFI in this study. Findings from previous studies are in agreement with our report (Shinefield et al., 1998; Miller et al., 2011; Vesikari et al., 2013) showing that two or more vaccines could be administered safely during the same vaccination session. Administering a child with several vaccines during the same visit offers some advantages, both in terms of an earlier onset of protection and in terms of saving costs and time. Moreover, the administration of multiple vaccines can represent a valuable measure to reduce pain at time of vaccination (Saleh et al., 2017). Babies’ vaccination-related anxiety could be less significant when two or more vaccines are administered once than in more than one vaccination sessions (Dodd, 2003). Reducing the number of sessions provides benefits not only to infants, but also to parents, healthcare providers, office managers, and managed care administrators.

In terms of AEFI, our results are comparable with those published in the scientific literature over the past decades, both in adults (Alguacil-Ramos et al., 2016; Mentzer et al., 2018) and in children (Danova et al., 2017; Lopes et al., 2018; Mentzer et al., 2018). The adverse events related to the use of vaccines occur at a very low frequency and are generally irrelevant when compared to risks associated to non-vaccination (Aps et al., 2018). The overall literature provides no substantial indication of a causal relationship between immunization and the very occasional serious AEFI (Swedish Council on Health Technology Assessment, 2009). According to a 2014 study from the Centers for Disease Control and Prevention (CDC) (Whitney et al., 2014), vaccines have saved thousands of lives worldwide, in particular in children, preventing more than 300 million kids from getting sick only in United States. CDC also reported that the majority of AEFI (more than 90%) described in literature are generally not serious. A 2011 report from the National Academy of Medicine reviewed more than 1,000 vaccine studies and concluded that serious AEFI to vaccines are rare (Committee to Review Adverse Effects of Vaccines, 2011).

Another concern about vaccination safety refers to the presence of allergens, but as reported in our study, allergens could be considered safe and harmless (Matsumoto et al., 2017). To date, only few allergens in traces are currently present in human vaccines, and available evidences have already demonstrated their safety (Bastola et al., 2017). Data from the present study confirm that the presence of allergens in vaccines is not correlated with the onset of AEFI.

Focusing on Italy, the 2018 annual report on post-marketing surveillance of vaccines published by the Italian Medicines Agency (AIFA) showed a national rate of AEFI reporting of 11.1 per 100,000 inhabitants for all vaccines (mandatory and non-mandatory) (AIFA, 2018), while for Tuscany alone is of 8.0 per 100,000 inhabitants. According to our data, the actual rate for Tuscany is even lower (5.95 per 100,000 inhabitants). In fact, in our analysis, only AEFI reports associated with vaccinations performed during 2017 were evaluated. Instead, AIFA annual report contains also AEFI reports referred to vaccination administered several years before 2017. The other variables considered by AIFA in the annual report (i.e., sex, age, SOCs, preferred terms, etc.) are consistent with our results.

## Strengths and Limitations

This study has several limitations and strengths. First, its retrospective nature may have led to an underestimation of AEFI rate, since not all patients presenting an AEFI, even if serious, attend ED or spontaneously report the adverse event. However, considering that reporting all observed AEFI is compulsory for health professionals, and furthermore, data on AEFI reports in Tuscany were collected both through the regional spontaneous reporting system and two active pharmacovigilance projects, the issue of underreporting can be considered of relatively low relevance. Second, our analysis is based on AEFI reports that are affected by limits that include inaccurate and incomplete information, mainly related to lack of clinical data (Scavone et al., 2018a). Given that, we cannot always exclude the absence of information that was not listed in AEFI reports, and that might have influenced the clinical evaluation of each report (i.e., the lack of vaccination’s date or previous/current patient medical conditions which could affect the evaluation of causality assessment). Moreover, we have only Tuscan AEFI reports, which cannot be considered fully representative of the other Italian regions and, although our cases were all clinically evaluated, due to the large number of statistical tests performed the outcome of these tests should be regarded as descriptive. Given that the information on influenza vaccines administration is recorded in an administrative database different from those used for our analysis, we were not able to calculate the rate of AEFI to influenza vaccines in our sample.

Despite these limitations, this is the first retrospective analysis performed in Italy on potential predictors of AEFI seriousness in children over 1 year of observation. Moreover, focusing on AEFI reporting rate, since we evaluated AEFI related only to vaccination performed during 2017, our rate can be considered less influenced by the number of AEFI reports derived from the mass media influence on vaccination hesitancy observed in Italy before Law 119/2017 (introducing mandatory vaccination for 10 vaccines) was approved. We strongly believe that pharmacovigilance studies are a valid scientific tool, simple and inexpensive, allowing healthcare professionals to detect and better characterize AEFI in clinical practice. In fact, we were able to highlight vaccination safety, showing a very low rate of AEFI and demonstrating that, particularly in children, their seriousness is not related to the number of vaccines and strains/toxoids simultaneously administered.

## Conclusion

AEFI is a major concern, both regarding the public opinion as well as the healthcare politics, and it represents a challenge for healthcare professional and healthcare services. The present study provided new insights on the factors that might influence the risk of AEFI reporting in children (i.e., concomitant drugs, number of strains/toxoids simultaneously administered, and presence/absence of allergens). Based on our results, the risks associated with immunization do not justify vaccination avoidance, especially because, among the risks related to vaccines, non-vaccination could be considered the most important. In this context, novel and specific educational approaches are needed in order to transmit the importance to correctly understand scientific research on vaccine safety to the younger generations of citizens, who will be the parents of tomorrow.

## Data Availability

Datasets are available on request.

## Author Contributions

Study design was contributed by NL, GC, AB, and AV, with assistance from the rest of the authors. AB took the lead in data analysis, assisted by NL and GC, and data interpretation was performed by NL, GC, AV, CA, and PB, with assistance from the other authors. The manuscript was written primarily by NL and GC, with assistance from the other authors, and revised by MT, MR, RB, CR, ML, AM, SR, and FL. All authors approved the final version of the manuscript.

## Funding

This study was funded by a research grant from the AIFA (the Italian Medicines Agency), Rome, Italy, Tuscan County resolution DGRT 790/2016 All. C. The funder of the study had no role in the collection, analysis and interpretation of data, nor in the writing of the report, nor in the decision to submit the article for publication.

## Conflicts of Interest Statement

The authors declare that the research was conducted in the absence of any commercial or financial relationships that could be construed as a potential conflict of interest.
