# Supplementary material for: Vaccines Safety in Children and in General Population: A Pharmacovigilance Study on Adverse Events Following Anti-Infective Vaccination in Italy
Source: Front Pharmacol. 2019 Aug 30;10:948. doi: 10.3389/fphar.2019.00948 (PMC6728926; doi:10.3389/fphar.2019.00948)
Supplement: Supplementary file 1 [file Table_1.docx]

**Supplementary Table 1** - Most frequent SOC classes and AEFI (preferred terms), stratified according to reported seriousness.

|  | **Tot AEFI**  **n=570 (%)** | **Non-serious**  **n=431 (%)** | **Serious**  **n=139 (%)** |
| --- | --- | --- | --- |
| **10 most frequent SOC classes** |  |  |  |
| General disorders and administration site conditions | 267 (46.84) | 219 (50.81) | 48 (34.53) |
| Nervous system disorders | 67 (11.75) | 42 (9.74) | 25 (17.99) |
| Skin and subcutaneous tissue disorders | 62 (10.88) | 55 (12.76) | 7 (5.04) |
| Gastrointestinal disorders | 60 (10.53) | 37 (8.58) | 23 (16.55) |
| Musculoskeletal and connective tissue disorders | 30 (5.26) | 20 (4.64) | 10 (7.19) |
| Psychiatric disorders | 11 (1.93) | 7 (1.62) | 4 (2.88) |
| Infections and infestation | 11 (1.93) | 7 (1.62) | 4 (2.88) |
| Blood and lymphatic system disorder | 11 (1.93) | 7 (1.62) | 4 (2.88) |
| Eye disorders | 10 (1.75) | 8 (1.86) | 2 (1.44) |
| Respiratory, thoracic and mediastinal disorders | 9 (1.58) | 9 (2.09) | 0 |
| **10 most frequent AEFI (preferred terms)** |  |  |  |
| Fever | 100 (17.54) | 74 (17.17) | 26 (18.71) |
| Pain at site of vaccination | 30 (5.26) | 29 (6.73) | 1 (0.72) |
| Rash at site of vaccination | 30 (5.26) | 29 (6.73) | 1 (0.72) |
| Rash n.s. | 29 (5.09) | 25 (5.80) | 4 (2.88) |
| Oedema at site of vaccination | 27 (4.74) | 25 (5.80) | 2 (1.44) |
| Vomit | 23 (4.04) | 14 (3.25) | 9 (6.47) |
| Fatigue | 16 (2.81) | 11 (2.55) | 5 (3.60) |
| Erythema | 16 (2.81) | 16 (3.71) | 0 |
| Arthralgia | 15 (2.63) | 9 (2.09) | 6 (4.32) |
| Headache | 15 (2.63) | 12 (2.78) | 3 (2.16) |

**Supplementary Table 2** - Characteristics of all AEFI reports and each AEFI in the paediatric population.

|  | **0-15 months** | | | **16 months-12 years** | | | **12 -18 years** | | |
| --- | --- | --- | --- | --- | --- | --- | --- | --- | --- |
|  | **Non-serious**  **n=46 (%)** | **Serious**  **n=18 (%)** | **p-value** | **Non-serious**  **n=50 (%)** | **Serious**  **n=16 (%)** | **p-value** | **Non-serious**  **n=8 (%)** | **Serious**  **n=4 (%)** | **p-value** |
| **Age, months** |  |  |  |  |  |  |  |  |  |
| Median (IQR) | 6.50 (4.00 – 12.78) | 6.94 (4.70 – 12.00) | *0.935* | 72.00 (36.00 - 114.23) | 27.22 (17.87 – 63.12) | *0.020** | *167.00 (153.05 -185.09)* | *169.50 (163.00 – 192.49)* | *0.349* |
| **Sex** |  |  |  |  |  |  |  |  |  |
| Female | 13 (28.26) | 5 (27.78) | *0.530* | 22 (44.00) | 4 (25.00) | *0.239* | 5 (62.50) | 2 (50.00) | *0.679* |
| Male | 30 (65.22) | 13 (72.22) |  | 26 (52.00) | 12 (75.00) |  | 3 (37.50) | 2 (50.00) |  |
| *Missing* | 3 (6.52) | 0 |  | 2 (4.00) | 0 |  | 0 | 0 |  |
| **Ethnic group** |  |  |  |  |  |  |  |  |  |
| Caucasian | 28 (60.87) | 12 (66.67) | *0.573* | 33 (66.00) | 8 (50.00) | *0.516* | 5 (62.50) | 2 (50.00) | *0.679* |
| Others | 5 (10.87) | 3 (16.67) |  | 2 (4.00) | 1 (6.25) |  | 0 | 0 |  |
| *Missing* | 13 (28.26) | 3 (16.67) |  | 15 (30.00) | 7 (43.75) |  | 3 (37.50) | 2 (50.00) |  |
| **Causality** |  |  |  |  |  |  |  |  |  |
| Consistent (0) | 38 (82.61) | 11 (61.11) | *0.064* | 36 (72.00) | 7 (43.75) | *<0.001** | 8 (100.00) | 2 (50.00) | *0.091* |
| Inconsistent (1) | 1 (2.17) | 3 (16.67) |  | 1 (2.00) | 7 (43.75) |  | 0 | 1 (25.00) |  |
| Indeterminate (4) | 7 (15.22) | 4 (22.22) |  | 2 (4.00) | 0 |  | 0 | 1 (25.00) |  |
| Not classifiable (2) | 0 | 0 |  | 11 (22.00) | 2 (12.50) |  | 0 | 0 |  |
| **Preventability** |  |  |  |  |  |  |  |  |  |
| No | 41 (89.13) | 17 (94.44) | *0.512* | 49 (98.00) | 10 (62.50) | *<0.001** | 8 (100.00) | 4 (100.00) | *-* |
| Yes | 5 (10.87) | 1 (5.56) |  | 1 (2.00) | 6 (37.50) |  | 0 | 0 |  |
|  | **Non-serious AEFI**  **n=105 (%)** | **Serious AEFI**  **n=39 (%)** | **p-value** | **Non-serious AEFI**  **n=120 (%)** | **Serious AEFI**  **n=51 (%)** | **p-value** | **Non-serious AEFI**  **n=24 (%)** | **Serious AEFI**  **n=8 (%)** | **p-value** |
| **Concomitant drugs (not suspected)** |  |  |  |  |  |  |  |  |  |
| 0 | 98 (93.33) | 31 (79.49) | *0.007** | 118 (98.33) | 45 (88.24) | *<0.001** | 24 (100.00) | 8 (100.00) | *-* |
| 1 | 2 (1.90) | 6 (15.38) |  | 0 | 6 (11.76) |  | 0 | 0 |  |
| 2 | 5 (4.76) | 2 (5.13) |  | 2 (1.67) | 0 |  | 0 | 0 |  |
| **Tot strains/toxoids** |  |  |  |  |  |  |  |  |  |
| Median (IQR) | 4.00 (4.00 – 14.00) | 4.00 (4.00 – 4.00) | *0.002** | 5.00 (3.50 – 5.00) | 5.00 (1.00 - 10.00) | *0.590* | 8.00 (4.00 – 8.00) | 4.00 (3.50 – 4.00) | *0.001** |
| 1 | 5 (4.76) | 0 | *<0.001** | 8 (6.67) | 13 (25.49) | *<0.001** | 0 | 0 | *0.004** |
| 2-5 | 49 (46.67) | 37 (94.87) |  | 86 (71.67) | 18 (35.29) |  | 10 (41.67) | 8 (100.00) |  |
| 6-9 | 11 (10.48) | 0 |  | 10 (8.33) | 6 (11.76) |  | 14 (58.33) | 0 |  |
| 10-13 | 10 (9.52) | 0 |  | 10 (8.33) | 5 (9.80) |  | 0 | 0 |  |
| 14+ | 30 (28.57) | 2 (5.13) |  | 6 (5.00) | 9 (17.65) |  | 0 | 0 |  |
| **Presence of allergens (in traces)** |  |  |  |  |  |  |  |  |  |
| Yes | 49 (46.67) | 2 (5.13) | *<0.001** | 53 (44.17) | 24 (47.06) | *0.728* | 14 (58.33) | 2 (25.00) | *0.102* |
| No | 56 (53.33) | 37 (94.87) |  | 67 (55.83) | 27 (52.94) |  | 10 (41.67) | 6 (75.00) |  |
| **Seriousness** |  |  |  |  |  |  |  |  |  |
| Death | - | 0 | *-* | - | 0 | - | - | 0 | *-* |
| Persistent or significant disability/incapacity | - | 2 (5.13) |  | - | 6 (11.76) |  | - | 0 |  |
| Congenital abnormalities/birth defects | - | 0 |  | - | 0 |  | - | 0 |  |
| Hospitalization or prolongation | - | 31 (79.49) |  | - | 32 (62.75) |  | - | 6 (75.00) |  |
| Life-threatening condition | - | 0 |  | - | 0 |  | - | 0 |  |
| Other clinically relevant conditions | - | 6 (15.38) |  | - | 13 (25.49) |  | - | 2 (25.00) |  |
| **Outcome** |  |  |  |  |  |  |  |  |  |
| Complete resolution | 50 (47.62) | 24 (61.54) | *0.004** | 51 (42.50) | 18 (35.29) | 0.001* | 17 (70.83) | 6 (75.00) | *0.039** |
| Improvement | 24 (22.86) | 2 (5.13) |  | 18 (15.00) | 11 (21.57) |  | 4 (16.67) | 0 |  |
| Still unresolved | 18 (17.14) | 2 (5.13) |  | 12 (10.00) | 16 (31.37) |  | 0 | 2 (25.00) |  |
| Resolution with sequelae | 0 | 0 |  | 0 | 0 |  | 0 | 0 |  |
| Unchanged/worsened reaction | 2 (1.90) | 0 |  | 0 | 0 |  | 0 | 0 |  |
| Death | 0 | 0 |  | 0 | 0 |  | 0 | 0 |  |
| *Missing* | 11 (10.48) | 11 (28.21) |  | 39 (32.50) | 6 (11.76) |  | 3 (12.50) | 0 |  |
| **Interactions (DDIs + VDIs + VVIs)** |  |  |  |  |  |  |  |  |  |
| Not applicable (1 treatment) (99) | 81 (77.14) | 30 (76.92) | *0.341* | 106 (88.33) | 37 (72.55) | <0.001* | 24 (100.00) | 8 (100.00) | *-* |
| No (0) | 17 (16.19) | 9 (23.08) |  | 14 (11.67) | 7 (13.73) |  | 0 | 0 |  |
| Mild (1) | 0 | 0 |  | 0 | 0 |  | 0 | 0 |  |
| Moderate (2) | 2 (1.90) | 0 |  | 0 | 0 |  | 0 | 0 |  |
| Severe (3) | 5 (4.76) | 0 |  | 0 | 7 (13.73) |  | 0 | 0 |  |

**Supplementary Table 3** - Number of administered doses of vaccines for which no AEFI reports were collected in general population.

| Vaccine | ATC | N administered doses |
| --- | --- | --- |
| Yellow fever, live attenuated | J07BL01 | 2361 |
| Papillomavirus (human types 6, 11, 16, 18) | J07BM01 | 1485 |
| Hepatitis B, hepatitis A combinations | J07BC20 | 1450 |
| Typhoid, purified polysaccharide antigen | J07AP03 | 1188 |
| BCG vaccine | L03AX03 | 942 |
| Meningococcus A,C,Y,W-135, tetravalent purified polysaccharides antigen | J07AH04 | 822 |
| Encephalitis, Japanese, inactivated, whole virus | J07BA02 | 193 |
| Zoster, live attenuated | J07BK02 | 169 |
| Diphtheria-pertussis-poliomyelitis-tetanus-hepatitis B | J07CA12 | 124 |
| Encephalitis, tick borne, inactivated, whole virus | J07BA01 | 73 |
| Diphtheria-poliomyelitis-tetanus | J07CA01 | 55 |
| Diphtheria-hepatitis B-pertussis-tetanus | J07CA05 | 25 |
| Measles, combinations with rubella, live attenuated | J07BD53 | 22 |
| Measles, live attenuated | J07BD01 | 20 |
| Poliomyelitis oral, monovalent, live attenuated | J07BF01 | 14 |
| Diphtheria toxoid | J07AF01 | 13 |
| Rubella, live attenuated | J07BJ01 | 12 |
| Pneumococcus purified polysaccharides antigen and haemophilus influenzae, conjugated | J07AL52 | 11 |
| Measles, combinations with mumps, live attenuated | J07BD51 | 4 |
| Hemophilus influenzae B and hepatitis B | J07CA08 | 4 |
| Mumps, live attenuated | J07BE01 | 2 |
| Smallpox, live attenuated | J07BX01 | 2 |
| Typhoid, combinations with paratyphi types | J07AP10 | 1 |
| Rubella, combinations with mumps, live attenuated | J07BJ51 | 1 |

**Supplementary Table 4** - AEFI reports (non-serious and serious) stratified for each ATC according to the age groups (16 months-12 years and 12-18 years) of the National Vaccination Plan 2017-2019.

|  |  | 16 months-12 years | | 12-18 years | |
| --- | --- | --- | --- | --- | --- |
| Vaccine | **ATC** | **Non-serious** | **Serious** | **Non-serious** | **Serious** |
| Meningococcus C, purified polysaccharides antigen conjugated | J07AH07 | 1 | 0 | - | - |
| Meningococcus A,C,Y,W-135, tetravalent purified polysaccharides antigen conjugated | J07AH08 | 18 | 1 | - | - |
| Meningococcus B, multicomponent vaccine | J07AH09 | 8 | 3 | 1 | 2 |
| Pertussis, purified antigen, combinations with toxoids | J07AJ52 | - | - | 1 | 0 |
| Pneumococcus, purified polysaccharides antigen conjugated | J07AL02 | 2 | 4 | - | - |
| Tetanus toxoid | J07AM01 | 2 | 2 | - | - |
| Measles, combinations with mumps and rubella, live attenuated | J07BD52 | 8 | 3 | 0 | 2 |
| Measles, combinations with mumps, rubella and varicella, live attenuated | J07BD54 | 5 | 4 | - | - |
| Varicella, live attenuated | J07BK01 | 2 | 1 | - | - |
| Papillomavirus (human types 16, 18) | J07BM02 | 1 | 0 | 2 | 0 |
| Papillomavirus (human types 6, 11, 16, 18, 31, 33, 45, 52, 58) | J07BM03 | 0 | 1 | - | - |
| Smallpox, live attenuated | J07BX01 | - | - | - | - |
| Diphtheria-pertussis-poliomyelitis-tetanus | J07CA02 | 4 | 1 | 4 | 0 |
| Diphtheria-hemophilus influenzae B-pertussis-poliomyelitis-tetanus | J07CA06 | 0 | 1 | - | - |
| Diphtheria-hemophilus influenzae B-pertussis-poliomyelitis-tetanus-hepatitis B | J07CA09 | 3 | 0 | - | - |
